# Is it really a challenge to find positive attributes for international medical graduates predictive of success in family medicine residency?

**DOI:** 10.1186/s12875-023-02105-6

**Published:** 2023-07-14

**Authors:** Malcolm M. MacFarlane, Rosemary Pawliuk, Laura Blew

**Affiliations:** Society for Canadians Studying Medicine Abroad (SOCASMA), 22879-29B Avenue, Langley, BC V2Z 3B1 Canada

**Keywords:** International medical graduate, Bias, Marginalization, Residency selection

## Abstract

In their paper “Challenges with international medical graduate selection: finding positive attributes predictive of success in family medicine residency,” (BMC Prim Care 23(256):2–9, 2022) the authors report on their research into qualitative attributes that positively correspond to success in residency with the objective of assisting in the selection of International Medical Graduate (IMG) residents most likely to achieve success in family medicine residency. The authors found that positive predictors of IMG residents’ success were: presence of a positive attitude, proficient communication skills, high level of clinical knowledge, and trainability. The authors conclude that selecting IMG residents who possess these attributes will result in residents developing increased aptitudes for patient care. A careful reading of the paper raises a number of concerns. MacFarlane (Can Med Educ J 12(4):132–40, 2021) points out that IMGs are already marginalized in the residency selection process. Our concern is that this paper may contribute to this marginalization through a tone of negativity or bias against IMGs and the use of biased language throughout the paper that tends to cast IMGs as being inferior and somehow less well prepared for residency than Canadian Medical Graduates (CMGs). We argue that the proposed predictors are generic and equally relevant to both CMGs and IMGs. In focusing on these predictors in IMGs specifically, the paper appears to imply, without evidence, that IMGs are inadequate in the identified areas. After reviewing the paper’s references, the existing literature, and an analysis of language used, we conclude that IMGs are capable candidates for residency, and that the qualitative attributes outlined in the paper offer little utility for the selection of IMG residents relative to CMG residents.

In their paper “Challenges with international medical graduate selection: finding positive attributes predictive of success in family medicine residency,” [1] the authors report on their research into qualitative attributes that positively correspond to success in residency with the objective of assisting in the selection of International Medical Graduate (IMG) residents most likely to achieve success in family medicine residency. The authors found that positive predictors of IMG residents’ success were: presence of a positive attitude, proficient communication skills, high level of clinical knowledge, and trainability. The authors conclude that selecting IMG residents who possess these attributes will result in residents developing increased aptitudes for patient care. A careful reading of the paper raises a number of concerns. MacFarlane [2] points out that IMGs are already marginalized in the residency selection process. Our concern is that this paper may contribute to this marginalization through a tone of negativity or bias against IMGs and the use of biased language throughout the paper that tends to cast IMGs as being inferior and somehow less well prepared for residency than Canadian Medical Graduates (CMGs). We argue that the proposed predictors are generic and equally relevant to both CMGs and IMGs. In focusing on these predictors in IMGs specifically, the paper appears to imply, without evidence, that IMGs are inadequate in the identified areas. After reviewing the paper’s references, the existing literature, and an analysis of language used, we conclude that IMGs are capable candidates for residency, and that the qualitative attributes outlined in the paper offer little utility for the selection of IMG residents relative to CMG residents.

Suggestions of a negative bias regarding IMGs begin with the title of the paper: “Challenges with international medical graduate selection: finding positive attributes predictive of success in family medicine residency.” Whether intentional or not, the title suggests that it is challenging to select IMGs who will be successful in family medicine residency, or to find positive attributes in IMGs that might predict their success. In support of this proposition, the authors reference Andrew [3] asserting that Andrew found that IMGs consistently scored lower than CMGs on certification examinations of the College of Family Physicians of Canada (CFPC), and on In Training Evaluation Reports (ITERs). The authors also reference Mathews et al. [4] claiming that these authors identified shortcomings of IMGs on family medicine examinations as being of concern to medical educators.

On closer examination of these supporting references, it appears that the authors have misconstrued the referenced papers’ findings. Andrew reported that IMGs performed similarly to CMGs on ITERs but underperformed on CFPC examinations. It is important to note that Andrew’s study which reported on IMG performance in the IMG-BC family medical program dates to 2010. Interestingly, Thomson and Cohl [5] reported in 2011 that,

“Initially, the IMGs in the IMG-BC family medicine program generally performed as well as other residents in their program evaluations, but not as well in the national CFPC certification exam when compared with all BC residents across the various hospital sites. However, IMGs have progressively improved their performance and their results are now comparable with those of their Canadian-trained colleagues” (pg 114).

The improvement was attributed to “techniques for exam success”. This suggests that IMG performance on CFPC examinations is not clear cut, that there may be many factors that influence performance, including exam taking skills versus medical competence, and that performance may vary over time.

With regard to the Mathews reference, nowhere in the Mathews paper can we find a reference to medical educators being concerned regarding shortcomings of IMGs on family medicine examinations. Regarding IMG success rates, Mathews and colleagues reported 78.1% of IMGs received CFPC certification (i.e. passed exams). However, their study does not compare IMG outcomes on CFPC exams with CMG outcomes, therefore there is no way to determine whether a 78.1% success rate for IMGs is better or worse than the CMG success rate. Further, the authors’ analysis does not account for the fact that despite progress in examination design, exam-taking skills remain a factor that confound efforts at assessing competence. The authors’ conclusion that IMGs currently struggle with performance on family medicine examinations, and the implication that this reflects on competence, is not supported by the references provided.

Unfortunately, the difficulties with this paper run deeper. On page 2 of the paper, the authors note that, “An increasing percentage of IMGs have completed their medical degrees 5–9 years before beginning residency” then go on to suggest that “This gap in training could result in reduced recency of knowledge acquired in medical school and could play a role in IMGs struggling in examinations.” Entirely aside from the weakness of evidence provided by the authors to support IMGs as actually “struggling” in examinations, in a careful reading of the author’s own supporting reference, Mathew’s and colleagues found that there was not a significant difference between recent graduates and new graduates in passing the CFPC exam. There was a significant difference between recent graduates and new graduates in passing the Medical Council of Canada Qualifying Exam 2 (MCCQE2) but one which contradicts the authors’ hypothesis. In their discussion section, Mathews, et al. conclude that “Given that they have more clinical experience, it is not surprising that older graduates are more likely than their younger counterparts are to pass the MCCQE2 (an examination written after the first year of residency training); there is no difference between recent and older graduates’ performance on certification (eg, CCFP) examinations.“

“Blame for the failure of many IMGs on family medicine examinations” (Pg 2) on time since graduation is not supported by their own references. Also noteworthy is the authors’ use of language. The authors refer to “the failure of many IMGs,” yet the authors have failed to support their proposition that “many” IMGs have failed examinations. How many is “many”? The choice of wording implies a serious problem with IMG performance, yet this is not established. It is the use of language like this throughout this paper that gives rise to a perception of bias and negativity regarding IMGs.

Consider the following passages:

- “candidates can be selected that are better suited for the established education system.” (Pg 2) Are the authors implying that IMGs are not well suited to the established educational system?
- “more robust candidates can be selected and issues can be mitigated during residency, which will lead to family medicine practitioners who can provide patient care.” (Pg 2) Are the authors suggesting that IMGs are not robust candidates and that they have issues that mean they cannot provide adequate patient care?
- “Thus, if a candidate has been educated in a system that lacks adequate professionalism development, it will be challenging to institute such changes so late in training.” (Pg 6) The authors appear to be arguing that international educational systems generate physicians who lack professionalism and that, further, this lack of professionalism will be “challenging” to address in training. It is difficult not to see such a proposition as reflecting a negative bias against IMGs.

It may be argued that the authors’ key findings that residents’ success is enhanced by the presence of a positive attitude, proficient communication skills, a high level of clinical knowledge, and trainability are equally relevant to CMGs or any professional. In their conclusion, the authors acknowledge that “It is evident that the competencies revealed in this study are relevant to both IMGs and CMGs” yet they spend an entire paper trying to demonstrate that IMGs in particular may be challenged by inadequacies in these areas.

The biased language continues throughout the authors’ analysis of their four key indicators of success. With regard to trainability, the authors note that, “The attribute that most commonly associated to an IMG resident’s trainability was professionalism.” (Pg 3) Again, the authors appear to be implying that IMGs suffer from a lack of professionalism without offering any supporting evidence. The authors go on to further discuss professionalism stating that, “unprofessional residents present a strong association to lawsuits and adverse outcomes in their later careers.” (Pg 6) Such discussions in the context of a paper focusing on selection criteria for IMGs raises the spectre of IMGs as a source of unprofessional conduct, lawsuits, and adverse outcomes. Such implications can only have a negative impact on public and professional assessments of IMGs’ abilities.

The authors go on to link trainability with emotional intelligence, stating that, “Emotional Intelligence is understood as the ability to perceive, understand, and manage emotions in oneself and others.” (Pg 6) Again, in the context of a paper focused on characteristics that contribute to IMG success in residency, are the authors implying without evidence that IMGs lack emotional intelligence? The authors also highlight the ability to accept and integrate feedback as an important part of trainability. Concerningly, the authors suggest that “certain cultural norms may preclude proper assessment of this trait.” (Pg 6) Such an unsupported statement linking cultural norms with an inability to accept and integrate feedback is suggestive of a perspective that IMGs raised and educated in other cultures have difficulty accepting and integrating feedback. Such a perspective is highly questionable.

The above vignettes illustrate concerns regarding bias against IMGs reflected in this paper, and in at least some of the medical profession. A careful reader will notice other examples. The authors conclude that “Overall the methodology of this study is sound” and that, “All identified predictors were determined to correspond with IMG family medicine residents’ success in residency.” (Pg 6, 7) With respect, we disagree that the methodology of this study is sound. Aside from the lack of specificity of these predictors to IMGs, all interviews were conducted by the same interviewer, which introduces a level of bias that is not addressed in the paper. As the author notes, there may well be a sampling bias. With only 13 of 25 preceptors approached agreeing to participate, the question arises why others were reluctant, and whether some of those who did agree to participate may have chosen to do so because they wished to articulate negative experiences with IMGs. The authors do not appear to have explored the respondents’ reasons for deciding to participate in the study.

Finally, in the original paper [1], the study protocol is described as asking preceptors “reflective questions, which provoked them to draw on examples of exceptional and poor IMG performers they have taught and selected,” (Pg 2) yet there is no indication in Table 2 of the original paper [1] of what questions may have been used to provoke examples of poor IMG performers, and therefore no way of knowing whether interview questions may have provoked biased or negative responses from participants that may have contributed to the bias and negative tone observed in this paper. Also interesting in the study design is that the authors chose to limit the study’s focus to “examples of exceptional and poor IMG performers” and did not choose to expand the study to encompass “exceptional and poor CMG performers.” Such a methodology virtually guarantees that the study will find issues with IMG performance and as such, the study design inevitably led to the bias against IMGs that we have identified.

In conclusion, the attributes identified by the authors offer little to assist in the selection of IMG family medicine residents. Further, the implications throughout this paper of IMG inadequacy are disturbing and in contradiction to existing research and literature that demonstrates that IMGs are capable residency candidates and that IMG patient outcomes are equal to or superior to those of CMGs or North American trained physicians. [6, 7] The authors state that “IMG needs are not well defined” however we would argue that this is not true. Numerous authors have described the challenges that IMGs face in succeeding in residencies in Canada. [8–11] According to Najeeb and colleagues [11] one of the major challenges faced by IMGs is “discrimination because of negative labelling as IMGs.” Unfortunately, this paper offers little to address the needs of IMGs identified in the literature.

## Data Availability

Not applicable.

## Declarations

### Prior publication

No prior publication.
